# Data mining for psychological profiling of track and field athletes and runners

**DOI:** 10.3389/fpsyg.2024.1518468

**Published:** 2025-01-28

**Authors:** Cristina Sanz-Fernández, José Luis Pastrana Brincones, Julen Castellano, Rafael E. Reigal Garrido, Diego Arvizu-Lozoya, Antonio Hernández-Mendo, Verónica Morales-Sánchez

**Affiliations:** ^1^Department of Education Sciences, Universidad de La Rioja, Logroño, Spain; ^2^Department of Social Psychology, Social Work and Social Services and Social Antrophology, Universidad de Málaga, Málaga, Spain; ^3^Department of Physical and Sport Education, Universidad del País Vasco, Vitoria-Gasteiz, Spain; ^4^Universidad Autónoma de Nuevo León, San Nicolás de los Garza, Mexico

**Keywords:** track and field, data mining, clustering, mixed method, specialties

## Abstract

Psychological factors in sports have been widely studied in scientific literature. However, only a few studies have used data mining techniques for athletic profile analysis. The main goal of this study was to analyze motivation, self-confidence, flow, and psychological skills in athletics to build differentiated profiles through clustering techniques. The sample size was 470 participants (ages 14–70 years old; *M* = 32.1; SD = 13.5). The Sports Motivation Scale (SMS), Task and Ego Orientation in Sport Questionnaire (TEOSQ), Self-confidence in Sport Questionnaire (CACD), Flow Dispositional Scale-2 (FDS-2), and Psychological Inventory of Sport Performance (IPED) were used to analyze the psychological profile of the sample. A data clustering analysis was carried out to check the study’s purpose. Results show different behavior patterns according to specific profiles. Similarly, there have been differences between men and women, online and face-to-face participants, federated athletes and runners, categories, or sports disciplines. In conclusion, the understanding of each athlete’s psychological profile is essential to improve his/her performance. The results of this study could be used to implement changes and adjustments in athlete psychological training to run several intervention programs that focus on each group’s needs.

## 1 Introduction

Psychological factors in sports involve a set of mental characteristics and abilities presented by athletes (López-Roel and Dosil, 2019), which are crucial for sports performance. In recent decades, the number of investigations has increased, emphasizing the interest in this field (Lawless and Grobbelaar, 2015; Guíu and Leyton, 2019; Piepiora et al., 2019). This area of knowledge has grown significantly in both scientific and applied fields, highlighting different psychological constructs (Pastrana et al., 2019; Reigal et al., 2018). Nowadays, there is no doubt that it is necessary to look at the psychological skills related to sports performance to understand the behavior of athletes (Reigal et al., 2020).

Variables such as motivation, coping skills, visualization ability, flow state, and self-confidence have been extensively analyzed (García-Calvo et al., 2008; Jackson et al., 1998; Jackson et al., 2016; Cecchini et al., 2004; Sari et al., 2015; Koehn, 2013; Swann et al., 2015). However, these constructs have been studied many times in an isolated way. Earlier research has predominantly focused on individual psychological constructs, limiting a comprehensive understanding of athlete behavior. This study addresses these gaps by employing data mining and provides an integrative analysis of motivation, flow, and other psychological skills.

This approach may bias result interpretation, as it neglects other relevant variables, making it difficult to fully understand an athlete’s psychological profile (Pastrana et al., 2019). Therefore, assessing a wide set of psychological variables could be crucial to obtaining a global view of the psychology underlying each athlete.

One of the most studied psychological aspects of sports is the motivational level of athletes and the type of motivation they show. Motivation is defined as the interplay of cognitive, social, biological, and emotional variables that determine activity selection, practice intensity, persistence in the face of difficulties, and performance outcomes (Weinberg and Gould, 2010). For its evaluation, two of the most widely used theories in recent years have been the Self-Determination Theory (SDT) and the Achievement Goals Theory (AGT) (Ruiz et al., 2017; Sheehan et al., 2018; Gacio and Klay, 2019). SDT attempts to establish to what extent human behavior is self-determined or volitional, that is, to what extent people feel that their actions are the consequence of their own reasoned decisions or whether, on the contrary, they are experienced as the result of external pressures from the social context (Deci and Ryan, 2007). The Achievement Goals Theory assumes that how people judge their competence and define the success of their achievements influences their motivational patterns (Balaguer et al., 2007). Therefore, assessing these aspects is crucial to understanding the behavior of athletes in these contexts.

The perception of self-confidence is considered another of the most important variables for the athlete’s performance (Molina et al., 2017; Tomé-Lourido et al., 2019). Self-confidence in sports is the belief or degree of certainty an athlete has about his or her ability to succeed in his or her sport (Feltz, 2007; Feltz, 2007). Therefore, this variable is essential when evaluating the psychological profile of the athlete, which is appreciated by the amount of research conducted on this psychological variable (Weinberg and Gould, 2010). In this sense, self-confidence has been related to track and field and has been combined with different variables that have an impact on performance, such as anxiety (Jaenes-Sánchez et al., 2012) or self-esteem (Ruiz et al., 2017). More experienced athletes generally have tighter self-confidence and less anxiety before competition, making their personal assessment better (Feltz, 2007). Most studies on self-confidence coincide in understanding it as one of the variables most associated with pre-competition emotions (Hernández-Mendo, 2003).

Also, related to the challenges that the athlete has to face and the resources he/she possesses to carry them out, one of the psychological variables that has been most studied in recent years is flow (Jackson and Marsh, 1996; Jackson and Eklund, 2002; Stavrou et al., 2007). It refers to a state of concentration that is so focused that it keeps the person experiencing it in a state of total absorption in the activity, which provides them with genuine enjoyment, even though that activity is difficult or dangerous (Jackson et al., 2016). However, it is important to understand how different athletic populations experience the state of flow during sports performance due to the intrinsic characteristics of this state (Jackson and Csikszentmihalyi, 2002). Flow has been investigated in some endurance sports, including long-distance and ultra-long-distance track and field events, finding that the feeling of flow is related to the psychological well-being of athletes (Jackson et al., 1998) and different variables such as those explained above (Stavrou et al., 2007; Fernández et al., 2013). However, there is not as much research on the differences in the flow perception between different sports specialties.

In recent years, some authors have pointed out that analyzing a wide range of psychological variables essential for performing adequately in sports is necessary. To this end, many researchers have focused on the study of the so-called psychological skills, which have been an approach to a holistic and complete study of the psychological profile in sports. The concept of psychological profiling gathers the main skills of athletes who have succeeded in their sport, developing their sporting abilities to the maximum (Da Silva et al., 2018). Different instruments have been developed to evaluate it. For example, the Psychological Inventory of Sport Performance [IPED; (Williams, 1991; Hernández-Mendo, 2006)] comprises 42 items organized into seven factors: self-confidence, negative coping control, attentional control, visual-imaginative control, motivational level, positive coping control, and attitudinal control. Both are useful instruments that can be used depending on the research goals and the structure of skills that best fit the approach of the study. However, it should be noted that both leave out some psychological characteristics that can be interesting in different sports. Therefore, this research has considered extending the profile with the variables described above.

Specifically, in athletics, some studies have analyzed this sport’s psychological profile (e.g., Hernández-Mendo et al., 2014; Fernández-Macías et al., 2015; Ferraz et al., 2017; Ibrahim and Almoslim, 2016; McCormick et al., 2018; Zarauz and Ruiz-Juan, 2013), although few investigations still analyze a wide range of psychological skills in this sport. Some research has been made, especially in disciplines of endurance, but there is an important lack in other ones. For example, it has been studied self-efficacy (Zarauz and Ruiz-Juan, 2014), flow state (Fernández-Macías et al., 2015), or motivation (Gayton et al., 1986). In Spain, most research related to psychological constructs in athletics has been carried out with participants in the master’s category (over 35 years of age) (McCormick et al., 2018; Zarauz and Ruiz-Juan, 2013). There are studies in race walking (Hamer et al., 2002), marathons (Nieto and Olmedilla, 2001; Jaenes, 2003), or some throws (Jaenes et al., 2005) in which certain psychological variables related to the profile of the athlete have been analyzed. Several authors agree that psychological skills are one of the most influential factors in sports performance (González et al., 2019; García-Naveira, 2016), so their training is not only possible but also essential.

Even though there are limited studies on psychological profiling in athletics, some authors suggest the existence of a distinct psychological profile for this sport, as well as variations among athletes based on factors such as competitive level (Nicholls and Ntoumanis, 2010), age category (Beckford et al., 2016), or specialty (Boccia et al., 2018). Identifying these differentiated profiles is challenging due to the need to process large amounts of data. To address this, analytical techniques such as data mining can be employed. For instance, researchers such as Pastrana et al. (2019) and Filgueira (2015) discovered differences in psychological variables among athletes in other sports using data mining techniques. The findings of these studies highlight the effectiveness of data mining algorithms as a valuable tool for psychological profiling research. Thus, data mining presents a promising approach for analyzing psychological profiles in athletics. Given the limited research in athletics and the importance of analyzing psychological profiles in sports, the primary objective of this study was to evaluate various psychological characteristics—namely motivation, self-confidence, flow, and psychological skills (as assessed by the IPED)—in a sample of athletes using data mining techniques. Overall, this research aims to identify distinct psychological profiles in athletics and emphasize their practical implications for enhancing training and designing psychological interventions tailored to specific athletic specialties.

## 2 Materials and methods

### 2.1 Design

This research uses an exploratory, cross-sectional, and non-experimental design (Franquelo et al., 2021).

### 2.2 Participants

The sample comprised 470 participants (266 men and 204 women) who were selected randomly and assured of their data’s confidentiality and anonymity. Among them, 241 completed the questionnaire online, while 229 completed it on paper. All participants were engaged in various specialized athletic disciplines. Specifically, 148 were prominent in distance running, and 322 were registered athletes. Within this group, 95 were sprinters and/or hurdlers, 143 were middle-distance and/or long-distance runners, 81 were specialists in track events (jumps and throws), and 9 were decathletes or heptathletes. Their ages ranged from 14 to 70 years (*M* = 32.1; SD = 13.5). These ages were categorized into three groups: under 18 (from 14 to 18 years), senior (from 19 to 35 years), and master athletes (over 35 years).

### 2.3 Instruments

Participants filled out a preliminary questionnaire to collect sociodemographic data relevant to the research. Within this, data was collected on the following variables: age, sex, type of athletics practiced (running or federated), and specialty. In addition, they took a battery of five questionnaires, including the following:

The Sports Motivation Scale [SMS; (Franquelo et al., 2021)] measures self-determined motivation and is structured into 28 items and seven dimensions. This instrument is answered with a Likert-type scale ranging from 0 (strongly disagree) to 10 (strongly agree). Cronbach’s alpha values for each subscale have values of intrinsic motivation to get stimulation (“because I like to discover new training techniques”): *α* = 0.76, intrinsic motivation to get things (“for the thrill I feel when I’m totally into the sport”): *α* = 0.83, intrinsic motivation to know (“because of the satisfaction of knowing more about this sport”): *α* = 0.80, identified regulation (“because in my opinion it is one of the best, in my opinion, it’s one of the best ways to meet people”): *α* = 0.68, introjected regulation (“because it is absolutely necessary to do sport to be fit”): *α* = 0.64, external regulation (“because it allows me to be valued by the people I know”): *α* = 0.74 and no motivation (“I do not know, I feel that I am not capable of succeeding in this sport”): *α* = 0.75.

Task and Ego Orientation in Sport Questionnaire [TEOSQ; (Ato et al., 2013)] is made of 13 items and consists of two scales measuring task orientation (7 items) and ego orientation (6 items). The TEOSQ items begin with the statement, “I feel more successful in sport when....” Subjects are asked to respond to the items using a 5-point Likert-type scale (1 = strongly disagree to 5 = strongly agree). The authors reported Cronbach’s alpha values of 0.80 for ego orientation (“I can do it better than my peers”) and 0.78 for task orientation (“I learn a new task by making an effort”).

Self-Confidence in Sport Questionnaire [CACD; (Balaguer et al., 2007)] assesses athletes’ self-confidence in competitive situations. It consists of 7 items with a Likert-type response format from 1 to 7 (1 = strongly disagree; 7 = strongly agree). Athletes are asked to respond to how they think and feel while immersed in a competition (e.g., “I always think I can win regardless of the opponent.”). The authors reported adequate internal consistency values of this instrument, with a Cronbach’s alpha of 0.90.

The Flow Dispositional Scale-2 [FDS-2; (García-Calvo et al., 2008)] assesses athletes’ flow through 36 items distributed into nine scales. All of these scales are answered on a Likert-type scale ranging from 1 (strongly disagree) to 10 (strongly agree) and correspond to the best-re-called experience. This scale showed a Cronbach’s alpha of 0.91 for total flow and for each of the subscales higher than 0.55. Specifically, the following values were obtained: challenge-ability balance (“I knew that my skills would enable me to meet the challenge the challenge I was facing”) (*α* = 0.66), action-attention fusion (“My attention was completely focused on what I was doing”) (*α* = 0.57), clear goals (“I knew what I wanted to achieve”) (*α* = 0.72), unambiguous feedback (“I was sure that at that moment, I was doing very well”) (*α* = 0.72), concentration on the task (“I had total concentration”) (*α* = 0.66), feeling of control (“I felt total control of my body”) (*α* = 0.69), loss of self-consciousness (α = 0.64), distorted sense of time (“the passage of time seemed to be different from normal”) (*α* = 0.65) and autotelic experience (“I found the experience very valuable and comforting”) (*α* = 0.73).

The Psychological Inventory of Sport Performance [IPED; (Williams, 1991; Hernández-Mendo, 2006)] is used to measure the performance of athletes in sport. It assesses different skills of the athlete’s competitive psychological profile. Consisting of 42 items answered on a Likert-type scale from 1 (almost never) to 5 (almost always). The internal consistency analyses yielded the following Cronbach’s alpha values: Self-confidence (“I see myself more as a loser than a winner during competitions”) *α* = 0.77; Negative coping control (“I get angry and frustrated during competition”) *α* = 0.76; Attentional control (“I become distracted and lose my concentration during competition”) *α* = 0.82; Visuo-imaginative control (“Before the competition, I imagine myself executing my actions and performing flawlessly”) *α* = 0.81; Motivational level (“I am very motivated to do my best in competition”) *α* = 0.75; Positive coping control (“I can maintain positive emotions during the competition”) *α* = 0.74; Attitudinal control (“During the competition, I think positively”) *α* = 0.76.

### 2.4 Procedure

The research was approved by the Ethics Committee of the University of the Basque Country (UPV/EHU). In addition, the guidelines of the Declaration of Helsinki (2013 revision), the recommendations of Good Clinical Practice of the EEC (document 111/3976/88 of July 1990), and the current Spanish legal regulations governing clinical research in humans (Royal Decree 561/1993) were followed.

Informed consent was obtained from participants prior to completing the questionnaire, either online or through the first page of the paper document. In both cases, the questionnaire took about 20–30 min to complete. Participant anonymity and data confidentiality were maintained throughout the study. In the online case, the questionnaire was completed via Google Forms, while in the paper case, data were collected from different popular races and federated competitions. Data were subsequently clustered using the k-means algorithm.

### 2.5 Data analysis

The data analysis was conducted employing clustering techniques. The WEKA program (Martínez-Romero et al., 2016) was utilized for this purpose. Clustering techniques encompass a set of unsupervised machine-learning methods for data clustering, with the K-Means algorithm being the most prominent. This is a multivariate technique aimed at partitioning data into groups that exhibit maximal homogeneity within themselves while being heterogeneous across groups. The K-Means algorithm demonstrates excellent scalability with data volume. When employing K-Means, the number of clusters (K) is predetermined, and the following steps are executed: initialization (random selection of centroid locations for the K clusters), assignment (assigning each data point to the nearest centroid), and updating (recalculating centroid position as the arithmetic mean of assigned data elements). These latter two steps are repeated iteratively until no further changes occur. This study performed two separate cluster analyses for each questionnaire type: one with two clusters (reflecting the inherent data tendency) and another with five clusters. The attributes (variables) taken into consideration were gender, athlete type (registered or non-registered), response format (paper or online), age category (under-18, senior, and master), and athletic discipline (middle-distance and/or long-distance, sprint and/or hurdles, track events –jumps and throws–, combined events). The justification for selecting two and five clusters lies in the inherent variability of psychological characteristics, as supported by preliminary statistical analyses and existing literature.

## 3 Results

### 3.1 Clustering for the total sample

Tables 1, 2 show clustering results for the whole sample, taking all the scores of the questionnaires analyzed. Table 1 show the default grouping found by the WEKA tool, and Table 2 shows the results of the analysis for five clusters grouping, taking as reference the five main variables of the study: gender, questionnaire response format, type of athlete, age category, and athletics sector.

**Table 1:** Whole sample clustering (by default grouping).

Attribute	Type participant	Cluster 0 (*n* = 273; 58%)	Cluster 1 (*n* = 197; 42%)
Format	Online	Online	Paper
Gender	Man	Woman	Man
Category	Senior	Senior	Master
Tipe	Federated	Federated	Runner
Sector	Middle/Long distance	Middle/Long distance	Runner
1-SCB	9	9.25	9
2-FAA	9	10	7.75
3-CG	10	10	9
4-CDF	8.5	9.5	8.5
5-TC	10	10	9
6-SC	8	10	8
7-LSC	10	10	8.75
8-DST	7	7	6.5
9-AE	10	10	9
10-IMS	10	10	8.75
11-IMT	10	10	8.5
12-IMK	10	10	8.25
13-EMID	7.5	8.5	7.5
14-EMIN	6.25	7	6.25
15-EME	1	1	2.75
16-NM	1	1	2.5
17-EO	1	1	1
18-TO	5	5	4
19-SC	3.71	3.71	4.86
20-SCI	20	22	20
21-NCC	18	15	13
22-AC	14	14	16
23-VIC	24	25	23
24-ML	23	23	20
25-PCC	24	26	23
26-ACC	22	26	21

SCB, skill-challenge balance; FAA, fusion of attention and action; CG, clear goals; CDF, clear and direct feedback; TC, total concentration; SC, sense of control; LSC, loss of self-consciousness; DST, distorted sense of time; AE, autotelic experience; IME, Intrinsic Motivation to Get Stimulation; IMT, Intrinsic Motivation to Get Things; IMKO, Intrinsic Motivation to Know; EMID, Extrinsic Motivation-Identified Regulation; EMIN, Extrinsic Motivation-Introjected Regulation; EME, Extrinsic Motivation-External Regulation; NM, No Motivation; EO: Ego Orientation; TO, Task Orientation; SC, Self-Confidence; ISC, IPED Self-Confidence; NCC, Negative Coping Control; AT, Attentional Control; VIC, Visuoimaginative Control; ML, Motivational Level; PCC, Positive Coping Control; ACC, Attitudinal Control.

**Table 2:** Whole sample clustering taking five clusters.

Attribute	Type participant	Cluster 0 (*n* = 139; 30%)	Cluster 1 (*n* = 112; 24%)	Cluster 2 (*n* = 81; 17%)	Cluster 3 (*n* = 89; 19%)	Cluster 4 (*n* = 49; 10%)
Format	Online	Online	Paper	Paper	Online	Paper
Gender	Man	Man	Man	Man	Woman	Woman
Category	Senior	Senior	Master	Master	Senior	Senior
Type	Federated	Federated	Federated	Runner	Federated	Runner
Sector	Middle/long distance	Sprint/Hurdles	Middle/long distance	Runner	Middle/long distance	Runner
1-SCB	9	10	8.5	9	8.75	7.75
2-FAA	9	10	8	8.75	9	7.5
3-CG	10	10	9	9	8.75	9.5
4-CDF	8.5	9.5	8.25	9	8.5	7.75
5-TC	10	10	8.75	9	9	7
6-SC	8	10	8	9	8	6.75
7-LSC	10	10	8.75	9.75	9	8
8-DST	7	10	6.5	7	5.25	5.75
9-AE	10	10	9.25	9.75	9	8
10-IMS	10	10	8.75	9	9.25	8.75
11-IMT	10	10	8.5	9	8	8.75
12-IMK	10	10	8.25	7.5	7	7.75
13-EMID	7.5	10	7.5	5.25	7	7
14-EMIN	6.25	10	7.25	6.25	5	8.5
15-EME	1	1	3	3.25	2	4
16-NM	1	1	2	2.5	2.25	1
17-EO	1	1	1	1.83	3.33	1.17
18-TO	5	5	4.29	4.43	4	4.29
19-SC	3.71	3.71	5	3.29	4.14	4.43
20-SCI	20	22	20	21	18	20
21-NCC	18	12	16	18	19	15
22-AC	14	15	16	17	14	16
23-VIC	24	30	18	23	24	18
24-ML	23	26	20	22	21	19
25-PCC	24	26	23	22	23	24
26-ACC	22	26	22	21	19	21

SCB, skill-challenge balance; FAA, fusion of attention and action; CG, clear goals; CDF, clear and direct feedback; TC, total concentration; SC, sense of control; LSC, loss of self-consciousness; DST, distorted sense of time; AE, autotelic experience; IME, Intrinsic Motivation to Get Stimulation; IMT, Intrinsic Motivation to Get Things; IMKO, Intrinsic Motivation to Know; EMID, Extrinsic Motivation-Identified Regulation; EMIN, Extrinsic Motivation-Introjected Regulation; EME, Extrinsic Motivation-External Regulation; NM, No Motivation; EO, Ego Orientation; TO, Task Orientation; SC, Self-Confidence; ISC, IPED Self-Confidence; NCC, Negative Coping Control; AT, Attentional Control; VIC, Visuoimaginative Control; ML, Motivational Level; PCC, Positive Coping Control; ACC, Attitudinal Control.

As can be seen in Table 2 (five clusters), Cluster 0 comprises 30% of the sample, representing senior men who responded to online questionnaires, are federated athletes, and specialize in sprints and/or hurdles. This athlete obtains very high scores in flow, is intrinsically motivated and task-oriented, and has high scores in self-confidence and good psychological skills.

Cluster 1 represents 20% of the sample and corresponds to the profile of a federated man, a middle-distance or long-distance running specialist, and older than 35 years. He gets a lower flow score than the previous group, but he gets high scores in this dimension. He is less motivated (intrinsically) than the previous group and scores higher in non-motivation. He is task-oriented and scores very low on ego-orientation. In addition, he has very high self-confidence (the highest possible score), and he has good psychological skills but lower scores than the previous group.

Cluster 2 gathers men over 35 who answered the questionnaire on paper and ran popular races. This cluster is characterized by individuals with high flow scores, albeit slightly lower than those in Cluster 0. In addition, they are generally less motivated and more ego-oriented than the other groups. Regarding task orientation, they are more task-oriented than Cluster 1 but less than Cluster 0. They have a lower level of self-confidence than the other clusters and good psychological skills. This cluster accounts for 17% of the total sample.

Cluster 3 comprises 19% of the sample and represents women aged 18–35 years who are federated and middle-distance and/or long-distance running specialists and completed the questionnaire online. She has a high perception of flow, with the exception of the dimension Distorted Sense of Time, where she scores considerably lower. In addition, she is less motivated than the other groups, less ego-oriented than the previous group, and has a high task orientation. She is also a person with high self-confidence and good psychological skills.

Finally, Cluster 4 corresponds to female runners of the senior category who answered on paper. Although their flow scores are generally lower than those of other groups, they score highly on clear goals. They are less motivated than other groups and have a high task orientation. They are very self-confident and have good psychological skills.

### 3.2 Clustering according to the scores on the different questionnaires

Once the analyses were carried out for the whole sample, other analyses were carried out separately for each questionnaire, taking the five clusters premise, corresponding to the expected athletics sectors in which the data were classified groups.

#### 3.2.1 Self-determined motivation

Table 3 shows the five clusters considering the scores obtained on the Sport Motivation Scale [SMS; (Ato et al., 2013)]. In this case, differentiated clusters correspond to 32, 28, 21, 11, and 8%, respectively.

**Table 3:** Whole sample clustering for self-determined motivation.

Attribute	Type participant	Cluster 0 (*n* = 152; 32%)	Cluster 1 (*n* = 131; 28%)	Cluster 2 (*n* = 99; 21%)	Cluster 3 (*n* = 51; 11%)	Cluster 4 (*n* = 37; 8%)
Format	Online	Online	Paper	Paper	Online	Paper
Gender	Man	Woman	Man	Man	Woman	Woman
Category	Senior	Senior	Master	Senior	Senior	Senior
Type	Federated	Federated	Runner	Federated	Federated	Federated
Sector	Middle/long distance	Middle/long distance	1.1.1 Runner	Middle/long distance	Middle/long distance	Sprint/hurdles
IMS	10	10	8	8.5	9.25	8.75
IMT	10	10	8.5	8.75	8	7
IMK	10	10	8.25	7.75	7	8.25
EMID	7.5	7.75	7.5	8	7	7
EMIN	6.25	5	6.25	7.25	8.75	8
EME	1	1	1	3.25	7	1
NM	1	1	1	2	1.5	1

IME, Intrinsic Motivation to Get Stimulation; IMT, Intrinsic Motivation to Get Things; IMKO, Intrinsic Motivation to Know; EMID, Extrinsic Motivation-Identified Regulation; EMIN, Extrinsic Motivation-Introjected Regulation; EME, Extrinsic Motivation-External Regulation; NM, No Motivation.

Cluster 0 corresponds to a senior category woman, federated and sprint and/or hurdles specialist who participated online. Among all the groups, this person is the most intrinsically motivated, with average scores in extrinsic motivation, except for extrinsic motivation-external regulation, where she scores very low, and no motivation, where she shows the lowest possible score.

Cluster 1 comprises men aged over 35 years who participate in popular races and who answered the questionnaire on paper. Their intrinsic motivation is high, but not as high as in the previous one. He also shows the lowest possible scores in No Motivation and External Regulation, with average scores in Extrinsic Motivation.

Cluster 2 comprises men aged between 18 and 35 years who are federated, middle-distance, and/or long-distance specialty and answered on paper. Those people are less intrinsically motivated than the previous groups and have higher scores on the extrinsic motivation and non-motivation dimensions.

In Cluster 3, scores are very high in intrinsic motivation to obtain stimulation, but they are also high in extrinsic motivation-external regulation, so it can be stated that this group of people is both intrinsically and extrinsically motivated. They are senior-category women who answered online and middle-distance and/or long-distance federated.

Cluster 4 comprises women aged 18–35 years who responded on paper, are federated athletes, and specialize in sprints and/or hurdles. Their scores are high across all dimensions of the questionnaire, with the exception of No Motivation and Extrinsic Motivation-External Regulation, where their scores are as low as those in Clusters 0 and 1.

#### 3.2.2 Achievement goals

Related to the Task and Ego Orientation in Sport Questionnaire [TEOSQ; (Balaguer et al., 2007)], the five clusters showed high scores in task orientation, as seen in Table 4.

**Table 4:** Whole sample clustering according to achievement goals.

Attribute	Type participant	Cluster 0 (*n* = 161; 34%)	Cluster 1 (*n* = 81; 17%)	Cluster 2 (*n* = 133; 28%)	Cluster 3 (*n* = 70; 15%)	Cluster 4 (*n* = 25; 5%)
Format	Online	Online	Paper	Online	Paper	Paper
Gender	Man	Man	Man	Man	Woman	Woman
Category	Senior	Master	Master	Senior	Senior	Senior
Type	Federated	Runner	Federated	Federated	Federated	Federated
Sector	Middle/long distance	Runner	Middle/long distance	Sprint/hurdles	Middle/long distance	Middle/long distance
EO	1	1	3	1.67	3.33	1.17
TO	5	5	4	5	4.57	4.29

EO, Ego Orientation; TO, Task Orientation.

Cluster 0 (34% representation) comprises master’s category male runners who participated online. Their scores are as low as possible in Ego Orientation and the highest in Task Orientation. Cluster 1 (17% of the sample), which corresponds to a male master category who answered on paper and middle-distance and/or long-distance federated, presents high scores for both orientations, while Cluster 2 (28% of the sample), typified by senior male, Sprint and/or Hurdles specialists who answered online, presents higher scores for Ego Orientation than Cluster 0 but lower than Cluster 1 while in Task Orientation they have the greatest possible score.

Cluster 3 (15% of the sample) comprised middle-distance and/or long-distance federated senior women who answered on paper. Scores in this cluster are high for both orientations. Specifically, this group scored highest in Ego Orientation and got the second highest score for Task Orientation in all groups.

Cluster 4 (5% of the sample; senior female paper respondents, mid-background and/or background specialists) presents low scores on Ego Orientation but high scores on Task Orientation.

#### 3.2.3 Self-confidence

Sports Self-Confidence Questionnaire results [CACD; (Balaguer et al., 1996)] are shown in Table 5. The scoring rate is between 3.14 and 5.14, i.e., there is medium-high self-confidence for all the participants.

**Table 5:** Whole sample clustering for the perception of self-confidence.

Attribute	Type Participant	Cluster 0 (*n* = 170; 36%)	Cluster 1 (*n* = 129; 27%)	Cluster 2 (*n* = 79; 17%)	Cluster 3 (*n* = 84; 18%)	Cluster 4 (*n* = 8; 2%)
Format	Online	Online	Paper	Paper	Online	Paper
Gender	Man	Man	Man	Woman	Woman	Woman
Category	Senior	Senior	Master	Senior	Master	Senior
Type	Federated	Federated	Federated	Federated	Runner	Federated
Sector	Middle/long distance	Sprint/hurdles	Middle/long distance	Middle/long distance	Runner	Middle/long distance
SC	3.71	3.71	5	5.14	3.14	3.29

Cluster 0 (36% of the sample) comprises sprinters and/or hurdlers. Senior category men who answered online presented an average self-confidence score, the third highest recorded score.

Cluster 1 comprises men aged over 35 years, long-distance and/or middle-distance running federated, taking the questionnaire on paper. They comprise 27% of the sample and are highly self-confident, with the second-highest score in the analyzed groups.

Cluster 2 (17% of the sample) has the highest self-confidence-scored people. They are middle-distance and/or long-distance federated senior women who answered the questionnaire on paper. Cluster 4(2% of the sample) comprises the same characteristics of people as cluster 2, scoring average rates in self-confidence tests but lower than clusters 0, 1, and 2.

Cluster 3 comprises women in the master category who participate in popular events and take the questionnaire online. Cluster 3 is 18% of the sample having average self-confidence scores, but those scores are the lowest in the sample.

#### 3.2.4 Flow

Results from the Flow Dispositional Scale [FDS-2; (García-Calvo et al., 2008)] can be seen in Table 6.

**Table 6:** Whole sample clustering for flow function.

Attribute	Type participant	Cluster 0 (*n* = 157; 33%)	Cluster 1 (*n* = 88; 19%)	Cluster 2 (*n* = 104; 22%)	Cluster 3 (*n* = 62; 13%)	Cluster 4 (*n* = 59; 13%)
Format	Online	Online	Paper	Paper	Online	Paper
Gender	Man	Man	Man	Man	Woman	Woman
Category	Senior	Senior	Master	Master	Senior	Senior
Type	Federated	Federated	Federated	Runner	Federated	Federated

SCB, skill-challenge balance; FAA, fusion of attention and action; CG, clear goals; CDF, clear and direct feedback; TC, total concentration; SC, sense of control; LSC, loss of self-consciousness; DST, distorted sense of time; AE, autotelic experience.

The first cluster represents 33% of the sample. It is made up of men aged between 18 and 35 years, sprint and/or hurdles specialists who answered the questionnaire online and have a very high perception of flow, getting the highest possible scores, with the exception of the dimension Clear and Direct Feedback, where the score is 9.5 out of 10 points.

The second Cluster is identified by a male of the master category who answers on paper, and he is middle-distance and/or long-distance specialty federated. His scores are generally lower than those of the people in the previous cluster, although he has a high perception of flow. The dimension in which they score lowest is Distorted Time Sense, while their highest score is in Action-Attention Fusion. They are 19% of the sample.

Cluster 2 is 22% of the whole sample, comprising a male who answers on paper. He is over 35 years old, and he is a runner. His perception of flow remains high, although he is the lowest scorer on the Feeling of Control dimension. However, his autotelic experience is very high.

The last two clusters have a representation of 13%. First, Cluster 3 comprises federated participants who are specialists in track events and answered the questionnaire online. Generally, they have high scores in all flow subscales, but they score lower than the other clusters. Their lowest scores are in Clear and Direct Feedback, and the highest scores are in Clear Goals and Loss of Self-Awareness. The last cluster is formed by federated women, middle-distance and/or long-distance running specialists who are over 35 years old. Women in this cluster get comparatively lower scores than in the other groups. The lowest score is in Distorted Sense of Time, as Cluster 2, where runners are also long-distance running specialists. Similarly, the highest score is in Clear Goals, as Cluster 1, where they are also middle-distance and/or long-distance specialists.

#### 3.2.5 Psychological skills

Finally, the groups obtained by clustering the Psychological Inventory of Sport Performance (IPED; [36; 37]) are analyzed in Table 7.

**Table 7:** Whole sample clustering for psychological skills.

Attribute	Type participant	Cluster 0 (*n* = 139; 30%)	Cluster 1 (*n* = 107; 23%)	Cluster 2 (*n* = 107; 23%)	Cluster 3 (*n* = 61; 13%)	Cluster 4 (*n* = 56; 12%)
Format	Online	Online	Paper	Paper	Online	Paper
Gender	Man	Woman	Man	Man	Woman	Woman
Category	Senior	Senior	Master	Master	Senior	Senior
Type	Federated	Federated	Federated	Runner	Federated	Federated
Sector	Middle/long distance	Sprint/hurdles	Middle/long distance	Runner	Middle/long distance	Middle/long distance
ISC	20	22	20	18	18	22
NCC	18	17	19	16	13	15
AC	14	15	14	17	11	16
VIC	24	30	18	23	24	26
ML	23	24	20	19	25	19
PCC	24	26	23	22	23	24
ACC	22	26	22	21	19	21

ISC, IPED Self-Confidence; NCC, Negative Coping Control; AT, Attentional Control; VIC, Visuoimaginative Control; ML, Motivational Level; PCC, Positive Coping Control; ACC, Attitudinal Control.

In this case, Cluster 0 (30% of the sample) comprises middle-distance and long-distance specialists, senior women, who took the questionnaire online. Their scores on psychological skills are high, especially in the Visuoimaginative Control dimension. Their lowest scores are on Attentional Control. This means they are people with a high capacity to imagine the competition and an average need to keep attention during the athletic event.

Cluster 1 (23% of the analyzed sample) is made up of men aged over 35 years answering on paper and middle-distance and/or long-distance federated. Scores in the different IPED dimensions suggest they have average psychological abilities, with people scoring between 14 and 23. Their best ability is Positive Coping Control, but Attentional Control is where they scored the lowest.

Cluster 2 comprises master’s category men, runners answering on paper. They are 23% of the sample having an average psychological ability. Negative Coping Control is the dimension where they score the lowest, but they score the highest on Visuoimaginative Control. Furthermore, people in this cluster scored the highest in attentional control.

Cluster 3 comprises senior women, middle-distance and/or long-distance running specialists answering the questionnaire online. They are 13% of the sample and get average scores on psychological skills. In general, it can be said that their scores are lower than the others, getting the lowest score on the attentional control dimension, but they got the highest score on the motivational level.

Finally, Cluster 4 is 12% of the sample and comprises senior category women, federated, middle-distance, and/or long-distance specialists answering on paper. Their best psychological skill is the Visuoimaginative Control, and the worst is the Negative Coping Control.

## 4 Discussion

This research aims to examine the psychological profile (consisting of motivation, self-confidence, flow, and psychological skills) in athletics using data mining techniques, specifically clustering. The outcomes from the comprehensive analysis of the entire sample underscore the intrinsic clustering tendency within the data, elucidating the presence of two discernible groups characterized by analogous scores across diverse subscales and questionnaires. These identified clusters delineate participants into distinctive categories. This demarcation in clustering suggests noteworthy divergences in questionnaire scores contingent on the examined sociodemographic variables. Therefore, clustering techniques enable establishing differentiated psychological profiles among athletes (Filgueira, 2015). The study unveiled distinct psychological profiles, offering practical insights into tailoring interventions to athletes’ specific needs.

Existing literature supports the idea that sociodemographic factors are pivotal in shaping athletes’ psychological profiles. The observed prevalence of women in the senior category within the first cluster resonates with studies highlighting gender-related differences in motivation and psychological responses to sports (Srivastava, 2014; Deaner et al., 2012). Moreover, the inclination of this cluster toward online questionnaire completion might reflect the growing influence of technology on data collection methodologies in sports psychology research (Ommundsen et al., 2006).

The distinct composition of the second cluster aligns with prior research on age-related variations in psychological attributes among athletes (Langan and Wheater, 2016). Specifically, the prevalence of male participants in the master category within this cluster might relate to age-related shifts in motivational orientations and coping mechanisms (Van Yperen et al., 2009). The preference for paper-based questionnaires in this group might also be associated with generational differences in technology adoption and comfort levels (Fry and Gano-Overway, 2010).

Firstly, in both clusters, the psychological profiles differ in terms of the five examined psychological constructs. The identification of distinct psychological profiles in both clusters, particularly in terms of the five examined psychological constructs, resonates with established research emphasizing the multifaceted nature of athletes’ psychological characteristics.

Motivation, a key determinant of athletic performance, was categorized into five discernible clusters, revealing complex relationships between gender and athletic specialization (Martin and MacDonell, 2012). These results align with existing research emphasizing the role of intrinsic motivation in sustaining athletes’ engagement and commitment to their sport (Martin and MacDonell, 2012). Moreover, the prevalence of task-oriented approaches among runners, especially those engaged in long-distance disciplines, is consistent with the literature highlighting the adaptive nature of task orientation in sports (Deci and Ryan, 1985).

The construct of Self-Determined Motivation demonstrated five distinct groupings based on how it was experienced. These results are consistent with previous literature, indicating differences in motivational profiles for athletics based on gender. Middle-distance and/or long-distance male specialists obtained higher scores than women in the same specialty for factors such as “Intrinsic Motivation to Achieve Things” (IMT), “Intrinsic Motivation to Know” (IMK), “Extrinsic Motivation-Identified Regulation” (EMI), and “No Motivation” (NM). Five different clusters were identified for achievement goals. However, the two of them shared similar sociodemographic characteristics. This finding suggests that the data may be better represented by four clusters, with one capturing a larger share of participants.

Additionally, it was observed that runners tend to be more task-oriented than middle-distance and/or long-distance federated athletes. Deci and Ryan’s study on Self-Determination Theory (SDT) provides a theoretical framework to understand the role of intrinsic motivation in sustaining athletes’ engagement and commitment. Our findings, revealing variations in intrinsic motivation across gender and specialization, echo the fundamental tenets of SDT.

Nicholls (Deci and Ryan, 1985) contributes to the discussion on task orientation in sports, highlighting its adaptive nature. Our observation of a prevalence of task-oriented approaches among runners, especially those engaged in long-distance disciplines, resonates with Nicholls’ perspective. Moving to Achievement Goals, the identification of five clusters with two sharing similar sociodemographic characteristics aligns with existing literature exploring the multidimensional nature of athletes’ goal orientations (Nicholls, 1989). This study is aligned with Deci and Ryan’s SDT, Nicholls’ study on task orientation, and Duda’s exploration of achievement goals adds depth to the interpretation of our results. By grounding our findings in established theories and empirical research, we contribute to the ongoing discourse in sports psychology, providing nuanced insights into the motivational and goal-setting dynamics within different athletic populations.

In terms of Self-Confidence, a critical factor in athletic success (Duda, 1989), distinct clusters exhibiting medium to high levels of self-confidence. Notably, the smaller cluster of senior women specializing in middle-distance or long-distance running, who responded on paper, displayed the highest self-confidence. The other three clusters represented middle-distance and/or long-distance male masters answering on paper, senior category sprinters men answering online, and female runners in the master’s category answering online.

This outcome highlights the significance of exploring both sociodemographic and methodological factors to fully understand athletes’ self-perceptions. Vealey’s extensive study on mental skills training in sports underscores the critical role of self-confidence in enhancing athletic performance. The identification of distinct clusters with varying levels of self-confidence aligns with Vealey’s emphasis on individual differences in athletes’ psychological characteristics. The heightened self-confidence among senior women engaged in middle-distance or long-distance running echoes findings in the literature, linking high self-confidence to successful athletic performance, particularly in endurance sports (Duda, 1989; Vealey, 2001).

Additionally, considering methodological factors, such as the mode of questionnaire administration (online vs. paper), adds a nuanced layer to our interpretation. The disparity in self-confidence levels across clusters might be influenced by demographic variables and the mode of response. While this observation may need further exploration, it aligns with existing research highlighting the impact of survey administration mode on athlete responses and self-report measures (Bandura, 1997).

Flow analysis shows a cluster that presents participants with a high perception of Flow, a strong sense of self-determination, a marked task-oriented approach, and proficient Psychological Skills compared to cluster 2. However, cluster 2 exhibits higher levels of Self-Confidence. Then, when examining the groupings obtained from the classification into five clusters, results show notable differences among the groups. One dominant cluster consists of male athletes taking surveys online, aged between 18 and 35 years, specializing in sprinting and/or hurdles. Their psychological profiles demonstrate very high perceptions of Flow and Self-Determined Motivation, a strong task orientation, low ego orientation, moderate Self-Confidence, and good Psychological Skills. The other clusters also show evident differences between sprinters and long-distance runners, and the way they responded to the questionnaire played a role in the clustering. The dominant groups exhibited heightened flow experiences, aligning with prior research emphasizing the positive relationship between flow and sports performance (Jackson et al., 2016). However, disparities in flow dimensions, such as distorted time perception, indicate the need for tailored interventions based on athletic specialty and demographic characteristics. In this research, the results align with previous findings that long-distance runners, including middle-distance specialists, do not experience distorted time intensely. However, gender differences were not found in this specialty, where men exhibit higher levels of flow overall compared to women.

The study’s findings align with existing literature on flow experiences in athletes, highlighting a predominant cluster characterized by high levels of flow, strong self-determination, and a task-oriented approach—attributes commonly associated with elite athlete profiles (Papinczak et al., 2016). Notable distinctions between sprinters and long-distance runners reinforce prior research linking athletic specialization to variations in flow experiences (Jackson and Csikszentmihalyi, 1999). The observed impact of questionnaire administration methods on clustering resonates with the broader literature emphasizing the significance of methodological considerations in flow research (Scanlan and Simons, 1992). Furthermore, the identified gender differences in flow levels, with men exhibiting higher overall flow, are consistent with existing studies (Jackson and Marsh, 1996), contributing to the broader understanding of flow experiences across athletic demographics.

Five distinct and relatively evenly distributed clusters were identified for psychological abilities. Senior women answering online and specializing in sprinting and/or hurdles exhibited the highest skills across the scale, except for “Control of Negative Coping” (NCC), “Attentional Control” (AC), and “Motivational Level” (ML). The observed exception in these three specific dimensions warrants careful consideration. These findings prompt a closer examination of the intricate interplay between psychological abilities and the demands of specific athletic disciplines. It is conceivable that the unique challenges posed by sprinting and hurdling events may necessitate distinct coping mechanisms, attentional processes, and motivational drivers compared to other specialties. Comparisons with existing literature indicate the need for a nuanced understanding of psychological abilities in sports. Our findings reinforce the significance of gender and specialization in athletic performance. However, the nuanced differences in specific psychological dimensions emphasize the complexity and context-dependent nature of psychological profiles among athletes. Previous research has consistently emphasized the pivotal roles of coping strategies, attentional control, and motivation in influencing athletic outcomes (Swann et al., 2015; Smith et al., 2018). Nonetheless, our study’s observed variations across diverse sports disciplines suggest a need for more in-depth exploration into the sport-specific nuances of psychological factors. Further research should delve into these sport-specific nuances to enhance our understanding of the multifaceted nature of psychological factors in athletic performance. These findings highlight the utility of data mining in sports psychology, particularly for creating tailored training programs informed by psychological profiling.

However, it should be noted that low sample equity in terms of sociodemographic variables is a limitation of this study. A more balanced sample across athletic sectors could have provided more robust results. Despite this limitation, consistent results were obtained, indicating the influence of different sociodemographic variables on the athletic psychological profile. This information could be valuable for implementing changes or improvements in athlete psychological training and their preparation and execution in competitions. Based on these findings, future research could focus on conducting similar studies with larger and more diverse samples and incorporating a greater number of variables. This could lead to the development of safe and effective psychological intervention programs for various types of athletes and provide individuals with tailored information for improving their sports performance.

In conclusion, identifying distinct clusters in psychological abilities offers valuable insights into the heterogeneity of athletes’ mental profiles. The prominence of senior women in sprinting and/or hurdles showcases the importance of considering sociodemographic and athletic specialization factors. The observed variations in specific dimensions warrant further investigation, emphasizing the need for a comprehensive understanding of psychological abilities in sports within a nuanced and context-specific framework.

## Data Availability

The data analyzed in this study is subject to the following licenses/restrictions: There are personal data of the participants. Requests to access these datasets should be directed to cristina.sanz@unirioja.es.
